# Diffusion-Limited Processes in Hydrogels with Chosen Applications from Drug Delivery to Electronic Components

**DOI:** 10.3390/molecules28155931

**Published:** 2023-08-07

**Authors:** Filipp V. Lavrentev, Vladimir V. Shilovskikh, Varvara S. Alabusheva, Veronika Yu. Yurova, Anna A. Nikitina, Sviatlana A. Ulasevich, Ekaterina V. Skorb

**Affiliations:** 1 Infochemistry Scientific Center, ITMO University, 191002 Saint Petersburg, Russia; alabusheva@infochemistry.ru (V.S.A.); yurowa.ver@yandex.ru (V.Y.Y.); nikitina@itmo.ru (A.A.N.); saulasevich@itmo.ru (S.A.U.); 2 Laboratory of Polymer and Composite Materials “SmartTextiles”, IRC–X-ray Coherent Optics, Immanuel Kant Baltic Federal University, 236041 Kaliningrad, Russia; vvshlvskh@gmail.com

**Keywords:** diffusion, hydrogel, drug delivery, encapsulation, controlled release fertilizer, wound healing, microswimmers, softrobotics, microrobotics

## Abstract

Diffusion is one of the key nature processes which plays an important role in respiration, digestion, and nutrient transport in cells. In this regard, the present article aims to review various diffusion approaches used to fabricate different functional materials based on hydrogels, unique examples of materials that control diffusion. They have found applications in fields such as drug encapsulation and delivery, nutrient delivery in agriculture, developing materials for regenerative medicine, and creating stimuli-responsive materials in soft robotics and microrobotics. In addition, mechanisms of release and drug diffusion kinetics as key tools for material design are discussed.

## 1. Introduction

Nature has had millions of years of evolution to obtain materials with the necessary properties. In this regard, using approaches implemented in nature is promising for developing new materials [1,2,3]. One of the key processes in nature is diffusion, which ensures the movement of molecules of a substance from an area of higher concentration to an area of lower concentration. Diffusion plays an important role in many processes, such as respiration, digestion, and nutrient transport [4,5,6]. When breathing, oxygen and carbon dioxide move through the membranes of the lungs, and the process itself is possible only due to a concentration gradient. In the process of digestion, the absorption of nutrients occurs through the intestinal membranes due to the difference in their concentration [7,8,9]. It is diffusion that provides our cells with nutrients and removes the products of their vital activity [10,11].

Thus, diffusion plays an important role in the life of organisms, ensuring the movement of various substances through membranes and maintaining the necessary concentrations of substances to sustain life. Thus, the study of diffusion features under multiple conditions can be used to simulate processes in living matter. Understanding diffusion is crucial for such diverse approaches as the design of new materials that can be used for prolonged controlled release [12,13,14,15], self-absorbable hydrogels for regenerative medicine [16,17], the creation of artificial muscles [18,19] and robots [20,21], and many others.

Hydrogels are a unique example of materials that allow control of diffusion and have found their application in wide fields such as encapsulation of drugs [22,23,24] and cells [25,26], analytical chemistry [27,28], fabrication of biosensors [29,30,31,32,33], and biomaterials for the bioactive agents’ delivery [34,35] as well as stimuli-responsive materials [36,37,38] and soft robotics [39,40].

All these applications mainly capitalize upon the hydrogel’s ability to restrict a solute’s diffusive movement [41]. Thus, understanding the parameters governing solute diffusion within hydrogels and how they affect diffusion is very important.

The aim of the present article is to review various functionalization approaches and resulting materials that may find practical applications from agriculture and regenerative medicine to soft robotics and microrobotics. In addition, mechanisms of release and drug diffusion kinetics as key tools for material design are discussed. In this review, we highlight the mechanisms of diffusion in hydrogels and move on to their practical applications for drug delivery, nutrient delivery in agriculture, developing materials for regenerative medicine, and creating stimuli-responsive materials in soft robotics and microrobotics.

## 2. Diffusion in Hydrogels

When discussing diffusion in hydrogels, we should regard its description of diffusion in liquids. As diffusion is driven by a gradient in Gibbs free energy or chemical potential, then from the point of view of thermodynamics, the driving potential of any levelling process is the growth of entropy [42]. At constant pressure and temperature, the role of such a potential is played by the chemical potential, which determines the maintenance of the flow of matter. In this case, the flow of particles of matter *J* is proportional to the concentration *C* and the chemical potential gradient (Equation (1)):

(1) $$J\sim -C{\left(\frac{\partial \mu }{\partial x}\right)}_{\rho ,Τ}$$

Using the decomposition of the chemical potential (Equation (2)) into powers of concentration, which is valid for gases and diluted solutions, it can be shown that the leading term in the expression for the particle flux is

(2) $$\mu =k_{B}T\ln C+\phi_{0}\left(T\right)+\phi_{1}\left(T\right)C+\ldots$$

Equation (3) indicates that the flux density of the substance *J* (measured, for example, in mol cm^−2^ s^−1^) is proportional to the diffusion coefficient *D* (cm^2^ s^−1^) and the concentration gradient.

(3) $$J=-D\frac{\partial C}{\partial x}\;$$

This equation expresses Fick’s first law. Fick’s second law relates spatial and temporal changes in concentration (diffusion equation, Equation (4)):

(4) $$\frac{\partial C}{\partial t}=\frac{\partial }{\partial x}\left(D\frac{\partial C}{\partial x}\right)$$

The diffusion coefficient *D* depends on the temperature. Usually in a wide temperature range, this dependence is the Einstein relation (Equation (5) [43]):

(5) $$D=\mu k_{B}T$$

However, Fick’s laws are applicable in the case of small concentrations *n* and concentration gradients −∇*n*.

The transfer processes of a multicomponent medium in the case of linear thermodynamic nonequilibrium systems described by the Lars Onsager model (Equation (6) [43]) are used:

(6) $$J_{i}=\underset{j}{\sum }L_{ij}X_{j}$$

Here, *J_i_* is the flow of the *i*-th component, *X_j_* is the thermodynamic force, and *L_ij_* is the kinetic coefficient matrix.

Unlike liquids, hydrogels can preserve the shape, and the water fluxes occur within certain static forms. Using porous gels makes it possible to significantly simplify the hydrodynamic picture and reduce the description of the spatial component of the processes to diffusion equations.

The diffusion in hydrogels is mainly considered to be Fickian. However, drug release often requires more complex models for a precise amount. Thus, recently some attempts at modeling complex cases where diffusion is influenced by swelling [44] or the diffusing molecule is sufficiently large, and the influence of the number of crosslinks strongly affects its progress, have been made [45]. It is assumed that diffusion in hydrogels is described, as in liquids, according to Equations (3) and (4).

The salute diffusion coefficient, *D*, is an important structural parameter that modifications of the polymer structure can control. Usually, it is assumed that D is independent of substance concentration (*c_i_*). This assumption is widely used when analyzing controlled release systems, but its validity is questionable [46]. Particularly for substance transport and release from polymeric systems, the interactions between substances are usually neglected. However, often, such interactions occur. For example, a protein/protein interaction significantly influences the release processes. Then, a more accurate expression of Fickian diffusion under these conditions would be (Equation (7)):

(7) $$\frac{\partial c_{i}}{\partial t}=\frac{\partial }{\partial z}\left\{D_{ip}\left(c_{i}\right)\frac{\partial c_{i}}{\partial z}\right\}$$

Such a concentration dependence can include the nonideal thermodynamic behavior of proteins and peptidic structures in terms of the activity coefficients of the protein. In the case of the membrane (reservoir)-type mechanisms, the concentration gradient across the polymer is quite large and almost constant for an extended release time. The first Fick’s law may be integrated over the thickness *δ*, assuming constant *J_i_* and *D_ip_*, to give Equation (8):

(8) $$J_{i}=\frac{D_{ip}K_{i}}{\delta }\Delta c_{i}$$

Here, ∆*c_i_* refers to the protein concentration dependence across the membrane and *K_i_* in a solubility-type coefficient which is included to account for sometimes significant changes in the protein solubility in the external (solution) phase and the polymer membrane. The dimensionless parameter *K_i_*, the protein partition coefficient, is calculated according to Equation (9).

(9) $$K_{i}=\frac{c_{in\;membrane}}{c_{in\;solution}}$$

The protein permeability coefficient, *P_i_*, is preferred to describe the overall protein permission through a polymer (Equation (10)).

(10) $$P_{i}=\frac{D_{ip}K_{i}}{\delta }$$

Obviously, protein permission, in addition to reasonable diffusion coefficient values, *D_ip_*, needs the partition coefficient to take large values. This requirement converts into important thermodynamic considerations for protein permeation.

As long as water makes up the bulk of the hydrogel and can freely pass through it, water permeation should be considered the main characteristic of this matter. Thus, its permeability is intensively used for better reaction control and process management.

The permeability coefficient *K* is usually calculated according to Equation (11):

(11) $$K=\frac{VL_{\eta }}{tA\Delta P}$$

In this formula, *V* means the volume of water (unit: mL), having a viscosity *η* (unit: poise), flowing through a sample of thickness *L* (unit: cm) and area *A* (unit: cm^2^) in a given time *t* (unit: s) under the pressure difference ∆*P* (unit: dyne/cm^2^), resulting in a value for *K* in square centimeters [47]

Usually, the permeability of the hydrogel depends on the average pore radius and its relation to the permeability coefficient according to Equation (12):

(12) $$r=\sqrt{\frac{8K}{S}}$$

The average pore radius *r* (in cm) depends on the permeability coefficient *K* and the specific water content *S*.

The equation is based on the following assumptions: (i) the water flows through parallel cylindrical capillaries of a circular cross-section; (ii) the water flow rate obeys Poiseuille’s law; (iii) all the capillaries are open to the surface, and there is no immobilized water lining in the pore walls.

Knowing the permeability coefficient *K*, we can calculate the diffusion coefficients by Equation (13):

(13) $$D=\frac{RTK}{\epsilon \bar{V}\eta }$$

In this formula, *D* is the diffusion coefficient (unit: cm^2^/s), *R* is a gas constant (8.314 × 10^7^ in ergs/°C·mole), *T* is the absolute temperature (unit: K), *K* is a permeability coefficient, *ε* is a fraction void volume (for which the specific water content is used, usually both values are approximately equal), *V* is the molar volume (18 cm^3^/mole), *η* is the viscosity (unit: 0.8937 × 10^–2^ poise). The substance transport processes are found to be governed predominantly by viscous flow in some diluted polymers, such as acrylamide. At the same time, diffusion becomes more important in more concentrated gels [47].

The thickness and stiffness of polymer chains affect diffusion in hydrogels by increasing the hydrodynamic drag on the solute molecule, which slows down the fluid near the polymer chain [48]. This effect is described in hydrodynamic models of the diffusion of solutes through hydrogels by the Stokes–Einstein equation (Equation (14)). It is believed that the solute moves at a constant speed in a continuum consisting of the solvent under the action of an applied force and resists the force of friction. The diffusion coefficient at infinite dilution *D*_0_ is expressed as:

(14) $$D_{0}=\frac{k_{B}T}{f\;}$$

in which *k_B_* is Boltzmann’s constant, *T* is temperature, and ƒ is the frictional drag coefficient. Hydrodynamic models of solute diffusion through hydrogels are therefore concerned with describing ƒ.

Additionally, obstruction theory suggests that the presence of impenetrable polymer chains causes an increase in the path length for diffusive transport, acting as a sieve and allowing passage of a solute molecule only if it can pass between the polymer chains [49]. However, the specific effects of thickness and stiffness on solute diffusion may depend on the size of the solute, the volume fraction of polymer present, and the composition of the polymer.

Dipolar molecular interactions and hydrogen bonds can affect the diffusion of solutes in hydrogels by creating a network of crosslinked polymer chains that restrict the movement of molecules [50]. Hydrogen bonds and dipole–dipole interactions between water molecules and polymer chains can create a high degree of hydration, resulting in a swollen hydrogel structure with a low diffusion coefficient [51].

Hydrogen bonding and dipole–dipole interactions can also affect the solubility of solutes in hydrogels. Hydrophilic solutes with polar functional groups can form hydrogen bonds with the hydrogel matrix, leading to increased retention and decreased diffusion [52]. On the other hand, hydrophobic solutes may be excluded from the hydrogel matrix due to unfavorable interactions with the polar groups in the polymer chains, resulting in low solubility and limited diffusion.

Overall, dipolar molecular interactions and hydrogen bonds play a significant role in determining the transport properties of hydrogels, including their ability to retain and release solutes. Understanding these interactions is critical for designing hydrogel-based systems for drug delivery, tissue engineering, and other biomedical applications.

At the same time, the previously employed Stokes–Einstein equation for predicting the diffusivity of solutes in hydrogels did not prove its accuracy, even when the mesh size was significantly larger than the drug size. It was shown that a more accurate diffusivity estimation is obtained when two diffusion modes contribute to the total probability: (i) via accessible volume voids and/or (ii) alongside water through the polymer mesh. The provided model explains the diffusivity more precisely and can be applied to explain the release within the drug carriers, even though the calculations included the solutes as hard spheres and the solutes and the polymer’s intermolecular forces are not considered.

Usually, closer attention is paid to a nanoobject’s diffusion through the bioinspired hydrogel mesh [53], showing the natural limitations of a purely mechanical approach. An attempt is made to describe another phenomenon of protein macromolecules amplified permeability in the hydrogel, where it was shown that a dynamic bonding to a matrix [54] and the crosslinking density [55] play a crucial role. On the other hand, some macroscopic diffusion models are also applied, which focus on the hydrogel as a specific object rather than a substance. The macroscopic factors used for shape-shifting can be temperature [51] or be a complex interplay between concentrations, temperature, and geometry. The linearity of diffusion pathways allows an exchange of temporal variables with the spatial surrogate. This approach has already found its implementation in the Belousov–Zhabotinnskiy reaction studies [56]. Experimental application of linear permeation is directly implemented in in situ detection of cell culture behavior with imposed drug concentration gradients [57].

## 3. Permeability of the Hydrogels as a Tool for Understanding Diffusion and Its Practical Application

Understanding their material properties, flexibility, interactions with solutes, and transport phenomena is essential to utilize hydrogels for various applications. In such cases, it is principal to study the permeability coefficient, which depends not only on the nature and physical and chemical properties of the substance diffusing through the hydrogel but also on the properties of the hydrogel itself. In particular, the polymer’s mesh size, shape, and percentage of crosslinking. We offer a look at them from the side of functionality and applications.

Recent advances in theoretical description have mainly focused on particular fundamental issues. Although the previously constructed theories describe the mechanistic noninteracting components, the new approaches are experiment-based and aim at the best fitting of experimental data. There are attempts at aggregated theories which combine the main approaches (hydrodynamic, free volume, and obstruction theory) to present a general predictive hypothesis [58]. However, the theory still describes the hydrogels mechanically.

Such simple behavior of solutes has found some practical applications. Firstly, they allow anisotropy-driven geometric planning for the specific pattern construction in hydrogels. This requires three main components: homogenous hydrogel with predictable diffusion pathways, reacting solutes, and spatially anisotropic setups for advanced diffusion control. Diffusion fluxes in homogenous hydrogels could be described on a purely geometric basis and allow the creation of spatially resolved structures. Different reacting substances lead to another final product. Single-component pH-controlled soluble salt to insoluble base transition in chitosan [59] or more complex processes such as polyelectrolyte coacervation or self-assembly processes [60] form continuous spatially distributed Liesegang rings. Periodic structures could also be formed by insoluble crystalline materials [61], self-assemblies [24], or metal complexation with subsequent nanoparticle growth [62]. Such spatially hindered diffusion control is reported to benefit significantly in controlling nonequilibrium rapid processes. In particular, the great advantage of the slow and constant supply of a reagent is implemented for predictable crystal growth. Controllable ion supply through hydrogels is reported to play a primary role in carbonate biomineralization [63,64]. Apart from biomineralization, hydrogels may be used for crystal shape perfection. Hydrogel-assisted crystal growth allows the production of perfectly shaped crystals. However, many of these crystals incorporate into the hydrogel matrix as macroscopic inclusions. That may greatly reduce the performance and quality of the crystal. The hydrogel is reported to affect the polytype stabilities for thermodynamically close modifications of Cu complexes [65].

Another way to create spatially limited setups is by introducing patterning or inhomogeneities into the hydrogel matrix. Directly creating complex shapes with preloaded hydrogels with further swelling and decomposition leads to miniaturized solid arrays [66]. At the same time, combined spatiothermal control of a crystallization direction has been utilized for heat-induced exotic exothermal crystallization [67]. Such approaches lead to the formation of preferred crystallization directions and allow self-induced anisotropic noninvasive structure formation. These structures would indeed find high demand in biomedical applications, for example, tissue engineering and design. The stiffness of hydrogel, derived from its composition, is also found to be applicable as a growth alteration factor [68].

The other useful feature of hydrogels is associated with the hydrophilicity of the scaffold. Hydrogel matrices may serve not only as an inert substrate but also as a fully functional composite component. Selective permeation of gels allows the formation of a protective layer. Polyacrylamide–alginate-based xerogels form a self-healing protective layer once the hydrophilic xerogel is rehydrated and polymer chains start diffusion-based rearrangement [69]. Wide-mesh polyacrylamide usage for protective hydrogels may remove gas microbubbles through microchannels without losing protective properties, making a water-permeable hydrogel an important part of water-splitting devices [70]. Another application is a stiff barrier of nonreactive hydrogels found due to dropping of the freezing point below 0 °C while the high water permeability remains unchanged. Hydrogels are implemented as an electrolyte carrier, more durable and easier to handle than conventional liquid electrolytes [71,72]. However, nonreactive organogels are even better suited for protection purposes and are proposed for highly reactive chemical storage such as of Li-organics [73].

**Figure 1:**
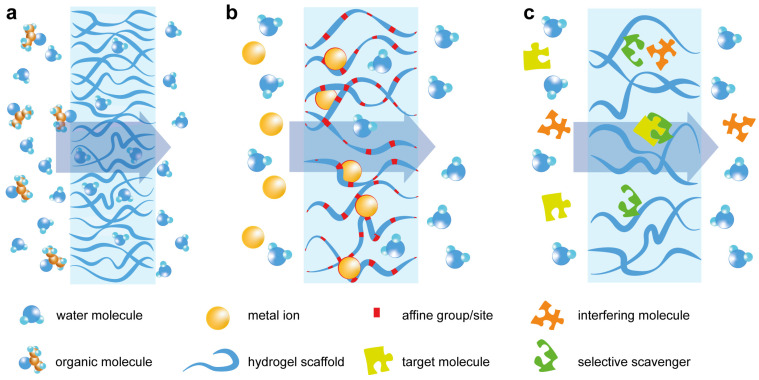
Permeable functional hydrogels for filtration and sensing purposes utilizing (**a**) local hydrophilic–hydrophobic interactions for organic removal; (**b**) introduction of metal-affine groups (red dots) into hydrogels; (**c**) introduction of selective groups for targeted chemical removal.

Further modification of hydrogels produces reactive materials with other applications, and the main application for functional or reactive gels is scavenging specific chemical substances from solvents. The main application of these materials nowadays is found in the filtration area (Figure 1a).

The small mesh size in hydrogels greatly enhances the influence of intermolecular interactions, and simple modification of the polyacrylonitrile polymer network with hydrophilic sodium polyacrylate parts leads to a tremendous rise in filtration selectivity for organic molecule removal [74]. Compared to classic surface-active membranes, macroscopic sizes of hydrogel-based membranes and interaction areas comodifying a core network with acid-resisting Kevlar fibers do not lead to sensitivity drop but dramatically enhance such a membrane’s utility [75]. Incorporating a self-healing agent into a selective hydrogel for water/organic splitting did not decrease the splitting selectivity [76]. Apart from additional modification, modern synthetic methods, such as host–guest organic synthesis [76], in situ ultrasonic matrix/filler homogenization with subsequent ultrasonic-assisted polymerization [77], or plasma-induced polymerization [78], are used for membrane improvement.

Metal ions are orders of magnitude smaller than the mesh sizes; however, their removal has a much higher priority on an industrial scale. Thus, there are some approaches for direct hydrogel modification, from enhancing the overall sorption capacity to metal-affine group addition and complex ion chelation (Figure 1b). A simple introduction of hyperbranched polyamide-amine improves the adsorption capacity of the chitosan/hyperbranched polyamide-amine/sodium alginate/calcium ion (CTS/PAMAM/SA/Ca^2+^) hydrogel due to the high number of reactive amino groups [79]. The addition of acrylamide to chitosan hydrogel is shown to give a high sorption capacity for anionic forms of Cr(VI) at low pH [80], and chitosan modified with alginate works for Cd and Pb ions even at neutral pH [81]. However, physical modeling describes the sorption as a Langmuir physical sorption with weak bonding to metal ions. Inorganic hydrogels tend to be more tolerant of ion exchange modifications and thus allow injection of affine metal centers for direct interaction with specific contaminants. Montmorillonite nanosheets were utilized as a robust template loaded with excess La^3+^ and Fe^3+^ for the quenching of As^5+^ [82].

Introducing metal nanoparticles (NPs) into the hydrogel matrix creates composites with enhanced properties. Immobilization of NPs through a chemical linkage prevents them from aggregating. It preserves the active surface of the particles for ligand adsorption while their functionality, such as magnetic susceptibility of Fe_3_O_4_ or light absorption of Au NPs, remains unchanged [83,84]. The introduction of CNTs expectedly enhances the sorption capacity for Pb^2+^ harvesting from solutions [85]. However, the abovementioned approaches lack selectivity despite their outstanding functionality.

Direct chemical interaction with solutes is a characteristic of another big group of hydrogels (Figure 1c), whose main area is specific substance removal where pure hydrophilic–hydrophobic interactions occur. A direct introduction of the isocyanate group by photoinduced copolymerization of 2-isocyanate ethyl methacrylate (ICEMA) and poly(ethylene glycol) methyl ether methacrylate (PEGMEMA) allows an easy functionalization with bioactive ligands to enable ligand-directed protein immobilization [86]. Highly selective protein-based hydrogels are great for controlled scavenging and release of selected substances, such as UO_2_^2+^, where the hydrogel thermal stretching induces uranyl binding protein to lose conformation, favorable for uranyl [87].

Observing a chemical interaction within a hydrogel opens the door to intelligent and robust detection systems. The hydrogel has to have good mechanical stability, be cheap, and have a good and selective response to qualify as a good candidate for a sensor platform. While the hydrogels act as a perfect matrix, MOFs have excellent optical properties. MOF@hydrogel composites are shown to be applicable for controlling food spoilage by smartphone-based trimethylamine detection by coupling with Ru@UiO-OH particles [88]. Chitosan-based hydrogels may be functionalized for detecting lanthanide ions, and PVA-chitosan–Tb composite was utilized for hypersensitive detection of nitroamines with photoluminescent response [89]. DNA hydrogels are the endpoint in the possible development of ultrasensitive sensors, as they act as a matrix and detector, and their selectivity in chemistry is unparalleled. Thus, detecting the selected gene using the CRISPR/Cas12a technology in a hydrogel using a glucometer was possible [90].

Hydrogels are an upcoming star among small-scale industrially viable functional materials as they best suit robust environments. They are more durable than thin films and easier to handle than liquid solutions, while their behavior is more predictable. The processes in hydrogels usually may be described based on geometry, mechanical assumptions, and chemical common sense, while the instrumentals for precise descriptions are constantly updating. The hydrogels find application in crystal growth, general water filtration, and selective chemicals’ scavenging as an ionic conductor and protective layer.

## 4. Diffusion-Controlled Drug Delivery by Hydrogel Systems

Diffusion plays a crucial role in hydrogel drug delivery, as it allows the controlled release of therapeutic drugs to targeted sites. In general, the majority of mechanisms of release are mediated by diffusion kinetics or hybrid processes within the polymeric drug carriers [47,91]. Usually, the carrier systems are presented by either reservoir or matrix systems, through which the encapsulated drug diffuses by a nonequilibrium concentration gradient.

The compartmentalization approach allows shielding of sensitive drug molecules with hydrogel capping shells and ideally provides the payload release directly at the specific site within a certain time and with an accurate amount of loaded drug. Hydrogel-based colloidal networks, or micro- and nanogels, made by crosslinking polymers, are biocompatible and capable of encapsulating small drugs and molecules into the polymer network, making them almost ideal platforms for delivering pharmaceuticals. However, the transportation and targeting processes, fulfilled by different stimuli and environmental conditions, remain critical. The efforts to overcome these challenges and improve the diffusivity of drugs using hydrogel-based drug delivery platforms are discussed in this section.

Functionalization and the introduction of compartments can bring about a diverse range of compositions with the possibility of spatial control and regulation of the diffused drug levels. Compartmentalization opens up new ways for variation in the inner structure of a complex hydrogel material, and in general, the compartmentalized compositions for drug delivery can be divided into three groups: (1) micellar/vesicular compartments [92], (2) droplet compartments [93], (3) multilayer compartments [94,95]. The presence of charged functionalities in the network is essential for achieving stimuli-responsiveness in hydrogels, a critical attribute for encapsulating and retaining theranostic cargo.

Drug-releasing platforms rely on the diffusion of therapeutics from macro-, micro-, and nanogels. Macroscopic drug-encapsulated hydrogels reach several centimeters, with the mesh diameter ranging from 5 to 100 nm [96]. The mesh diameter of the polymer network (or the mesh size, as the spacing between polymer chains is usually called) determines the amount and velocity of the encapsulated species diffused through the polymer matrix. Therefore, the mesh dimensions concerning the drug molecule size determine the diffusion limitation. More rapid diffusion occurs when the mesh size is larger than the encapsulated drug (r_mesh_/r_drug_ > 1) unless specific bonding forms between the molecule and the polymer chain [97]. When the mesh size value is close to the drug size (r_mesh_/r_drug_ ≈ 1), steric hindrance significantly impacts drug diffusion. For giant drug molecules compared to the mesh size (r_mesh_/r_drug_ < 1), steric hindrance leads to diffusion limitation, and the drug stay trapped within the network. Therefore, the molecule can only be released with the degradation of the network. This immobility of the drug until further net destruction is widely applied in stimuli-responsive hydrogels, which are discussed further in this section.

Currently, existing models are already able to predict the diffusivity of solutes through poly(ethylene glycol) (PEG) hydrogels by combining three distinct diffusion mechanisms: (a) hydrodynamic, (b) free volume, and (c) obstruction theory [58]. At the same time, the previously employed Stokes–Einstein equation for predicting the diffusivity of solutes in hydrogels did not prove its accuracy, even when the mesh size was significantly larger than the drug size. It was shown that a more accurate diffusivity estimation is obtained when two diffusion modes contribute to the total probability: (i) via accessible volume voids and/or (ii) alongside water through the polymer mesh. The provided model explains the diffusivity more precisely and can be applied to explain the release within the drug carriers, even though the calculations include the solutes as hard spheres and the solutes and the polymer’s intermolecular forces are not considered.

In general, the majority of mechanisms of release are mediated by diffusion kinetics or hybrid processes within the polymeric drug carriers [91]. Usually, carrier systems are presented by either reservoir or matrix systems, through which the encapsulated drug diffuses by a nonequilibrium concentration gradient. The diffusion rate may be increased by incorporating drugs into the polymer above the saturation point when the drugs remain undissolved within the matrix. For example, in-droplet precipitation of drugs before the polymer solidification or the lower critical solution temperature effect of thermoresponsive polymers and drug crystallization over the saturation level [98,99], amplifying the amount of the released drug at the disease site, may be applied. These diffusion mechanisms typically contribute to the passive transportation of drug carriers discussed below. In terms of operational mechanisms which determine diffusion modes, there are two primary modes of transporting micro- and nanogels to the intended destination, which are passive and selective transportation [100].

Passive transport of hydrogels is based on blood circulation, diffusion, and accumulation at the disease site. Passive transport is a process that does not require cellular energy expenditure and involves the movement of a substance across a membrane, following its concentration gradient. Typically, a phenomenon of the enhanced permeability and retention (EPR) effect [101,102,103] of tumor tissue increases the accumulation effect of passive transport systems (liposomes, nanogels, and macromolecules). These nanoparticles mostly rely on the degradation of the matrix and passive diffusion of entities through the cell membrane down the diffusion gradient, as well as on the enhanced leakage of newly formed blood vessels of the tumor tissues, allowing more accessible accumulation of carrier systems due to the EPR effect. The release of drugs through diffusions is determined by the mesh size, which typically falls within the range of hundreds of nanometers as an optimal size, approximately 100–200 nm [104]. The matrix’s degradation and swelling, whether time-dependent or triggered, can modify the retention effect of the drug carriers and, therefore, the efficiency of delivery systems.

Although many drug carriers’ issues, such as activation signals, control of the trigger in vivo, and materials’ complexity, have already been presented in various laboratory situations, clinical treatments still rely on passive diffusion systems [105]. Hydrogel-based platforms are often decorated with PEG hydrophilic blocks [106,107,108] to multiply the nano- and microgels’ blood circulation cycles due to better phagocytosis resistance and increased retention by the EPR effect [109]. However, although the FDA has approved other PEG-modified drug nanocarriers like liposomes that have been on the market since 1995 [110], only a few nanogel systems have been taken for clinical studies [111]. Currently, the limitations for their widespread application include the fast elimination of nanogels from the body by organs like the kidney, liver, and spleen. Therefore, further designing of the nanogel platforms relies on shape and size optimization and focusing on stimuli-responsiveness as a crucial attribute for more precise and rapid release of the theranostic cargo.

Selective transport for drug delivery is developed to control the diffusion and drug release profile. It refers to the ability of a drug delivery system to selectively transport drugs to a specific target site while avoiding nontarget sites. Several ways exist to improve drug delivery processes by amplifying mass transfer between drug-carrying vehicles and cells and reducing systemic toxicity by localized and controllable “on-demand” release [112]. Smart hydrogels may sense their microenvironment and react dynamically, mimicking the responsiveness of living organisms. Stimuli-responsiveness may be caused by two large groups of triggers [113]: (a) exogenous stimuli (temperature [114,115], light [116,117], ultrasound treatment [118,119], magnetic [120,121] or electric field [122,123]) and (b) endogenous stimuli (pH [124,125], enzyme concentration [126,127], or redox gradients [128,129]). Stimuli-activated release affects the properties, shape, structure, or integrity of the polymer matrix, which leads to hydrogel degradation by breakage of the polymer bonds or swelling of the hydrogel and drug release by solute adsorption. In the first case, the network serves as an actuator and degrades, and the mesh size increases, allowing the drugs to diffuse through the hydrogel. Typically, this type of release is determined by a complex mechanism of diffusion–erosion, when the polymer degrades, leaving large pores in the matrix that allow water to penetrate the polymer and intensify the release process. The balance between the diffusion rate and the degradation rate defines the nature of this type of release. When the diffusion rate exceeds the degradation rate, it leads to diffusion-controlled kinetics. When the degradation occurs before the drug release, it results in erosion-controlled kinetics [130]. Functional groups, transducing entities, and other environmental sensors play an important role in drug diffusion by matrix degradation. For example, pH-triggered release often includes carboxyl or amino groups, such as polydopamine (MPDA) nanoparticles, explicitly designed for the controllable release of tetracycline [131]. pH-dependent carriers are mainly chosen due to the pH variation within the human body from strong acidic to basic. This property may be applied to target specific tissues or organs, which is advantageous as it can provide a sustained release of the drug, leading to better therapeutic outcomes.

**Figure 2:**
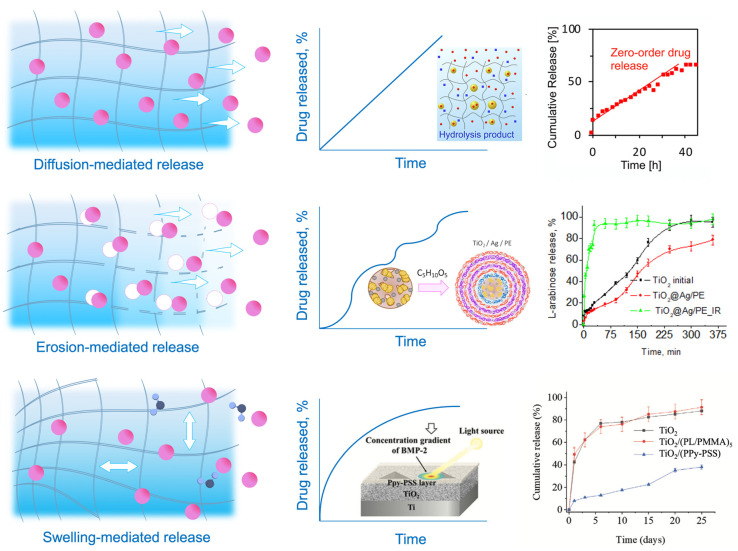
Mechanisms of release and drug diffusion kinetics. Drugs can diffuse with the liquid within the hydrogel [138] (diffusion-mediated release), through the increased mesh size by the matrix degradation [139] (erosion-mediated release), and through the increased mesh size by matrix expansion and water absorption [140] (swelling-mediated release).

Medical drugs are frequently released by enclosing them in a polymer that may expand upon fluid absorption and impact the diffusivity of the therapeutics. The drug is discharged from the domain’s boundaries, which may also increase in size as they absorb water or fluids. Swelling and deswelling of the hydrogel enable another diffusion-driven release mechanism by the increased porosity, allowing the encapsulated drugs to diffuse out of the drug carrier freely. Swelling and degradation may be actuated by various stimuli, including pH, temperature, magnetic field, etc. [132]. Specific polymer blocks may control the degree of swelling. For example, temperature-responsive polymers (poly(*N*-isopropyl acrylamide) (PNIPAM) [133], poly(*N*,*N*-dimethylacrylamide) (PDEAAm) [134], poly[2-(dimethylamino) ethyl methacrylate] (PDMAEMA) [135]) are designed in a unique way to stretch or shrink. These polymers usually include thermoactuating blocks such as hydrophobic (e.g., isopropyl) and hydrophilic (e.g., amide) groups, which allow the hydrogel to undergo a reversible phase transition, or swelling–deswelling, at a close to physiological temperature. An illustrative example is the thermoresponsive gelatin-based copolymer, designed to perform temperature-controlled prolonged release of T-lymphocyte-associated protein-4 for efficient and durable antitumor immunotherapy, or gingipain-responsive hydrogel (PEGPD@SDF-1) [136,137]. This effect allows the controlled release of drugs over a prolonged period, increasing their efficacy and reducing side effects. Figure 2 summarizes hydrogels’ described drug release mechanisms and compares their kinetics.

Overall, the diffusion properties of polymeric hydrogels play a critical role in determining the efficacy and duration of drug release. The diffusion of polymeric hydrogels can affect drug release by controlling the rate at which the drug molecules can diffuse out of the hydrogel matrix. The hydrogel matrix can act as a barrier to slow down the release of the drug, allowing sustained release over a more extended period. Diffusion maintains the balance between protecting the drug within the carrier from loss or degradation during transportation and its efficient on-demand release to the desired locus. The diffusion rate can be influenced by factors such as the size and charge of the drug molecules, as well as the hydrogel’s mesh size and swelling properties. External stimuli such as temperature, pH, and light can also affect the diffusion and release of drugs from the hydrogel matrix.

## 5. Practical Application of Limited Diffusion Kinetics in Regenerative Medicine

Apart from drug delivery, hydrogel matrices may adopt more crucial roles as a supporting basis for the growth of biological tissues in the replacement mode or even for embedding into future tissues and organs. Diffusion is still one of the key parameters in these processes. Therefore, hydrogels should be designed in such a way as to ensure the optimal pathways of transfer of nutrients to the cells inside the gel and metabolic products from the gel, as well as provide sufficient access to oxygen and other necessary elements. In hydrogels, diffusion can occur both inside the material and through its surface.

Due to the excellent biocompatibility and ability of polymer hydrogels to mimic the extracellular matrix structure, this regenerative material has found applications for biomedical purposes. In particular, this material can restore hard and soft tissues (skin, cartilage, and bladder) [141,142], serve as an adhesive and adhere to wet tissues [143], prevent bleeding as a new type of wound dressing [144,145,146,147,148,149], and serve as a barrier between tissues and material surfaces, protecting tissues or organs from aggressive external influences [150,151,152]. However, despite significant advances in the manufacture of multifunctional regenerative materials, some difficulties are associated with the limited diffusion capacity of hydrogels. The purpose of this section is to review recent advances based on limited diffusion mechanisms and design parameters for multifunctional hydrogels with controlled release of molecules.

**Figure 3:**
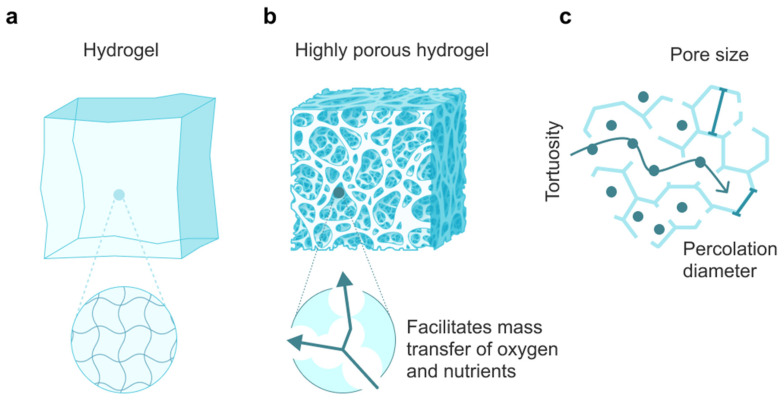
(**a**) Hydrogel structure; diffusion control strategies: (**b**) The effect of pores on diffusion in a hydrogel and (**c**) the effect of simulating the complexity of natural architecture on permeability.

By their nature, hydrogels are three-dimensional networks formed by hydrophilic crosslinked polymer chains (Figure 3a). Tissue regeneration is a complex process consisting of several stages, where the hydrogel is implanted into the damaged area of the tissue, after which it interacts with the surrounding cells and the extracellular matrix. In the process of attachment between the cells and the scaffold, a series of successive physicochemical reactions occur, where the hydrogel allows the cells to proliferate and differentiate into functional tissue, thereby being a platform. Generally, the theory of tissue regeneration in hydrogels is based on the principles of biomimicry and the delivery of bioactive molecules.

Some prospective approaches have been tested to control diffusion in hydrogels for regenerative purposes. For example, one can change the hydrogel crosslink density, polymer weight content, protein affinity, mesh size, and wall thickness to change the diffusion rate. One can also add various additives that speed up or slow diffusion (Table 1). External factors such as temperature and pressure also influence this process.

The kinetics of active ingredient release and nutrient and waste transport in tissue engineering depends on the rate of solute diffusion. Deceleration/accelerations can be caused by some factors, including various chemical and physical effects due to the attractive force between the solute and the hydrogel matrix. In this connection, a fundamental parameter for hydrogels in tissue engineering is the kinetics of degradation and regeneration / biomineralization processes [158]. A recent study described one strategy for controlling gelation in space and time for hydrogels [159]. The proposed regenerative material is photocrosslinked, oxidized, and methacrylate alginate hydrogel (POMA). In this system, alginate oxidation occurs, which demonstrates the possibility of using it to control kinetics by controlling physical properties. In particular, one of the adjustable properties is the elastic modulus, which can be controlled by changing the amount of low molecular weight alginate products formed during the oxidation reaction. The results of cell culture and encapsulation studies have demonstrated the excellent biocompatibility of POMA hydrogels, which, in combination with the ability to control degradation and biomineralization processes, show clinical potential as a scaffold in bone engineering.

Porosity is one of the key factors in transporting substances and oxygen to the cells within the gel, as this parameter indicates the volume of material available for cell infiltration or seeding (Figure 3b). Materials with higher porosity are considered favorable, allowing more system functional units. However, hydrogels of this type have a complex internal architecture since the pores, in addition to different sizes, can have varying geometry, curvature, and wall roughness [160]. The optimal mesh size for tissue regeneration is 20–125 µm [161] and for bone, 100–350 µm [162]. With a large mesh size, there is a decrease in intercellular interaction and, consequently, cell proliferation. The structure of the hydrogel is subject to the influence of the material’s surface properties. In particular, the penetration of substances into hydrogels with a mesh size of 25–50 μm was limited by the outer surface, while gels with mesh sizes of 50–100 and 100–150 μm allow the formation of tissue throughout the entire volume of the material [163]. The parameters of the pores can be altered to regulate the diffusion processes in the hydrogel, influencing cell permeability, the movement of nutrients, and metabolic waste. Research regarding the optimal size for cell motility remains relevant in this regard.

Consequently, most scientific research focuses on improving synthesis approaches with desired mesh sizes in hydrogels for regenerative medicine. In particular, a group of scientists led by Bin Duan and Seung-Hyun Ro proposed a simplified strategy for manufacturing porous hydrogels based on alginate (Alg) [164]. This material is structurally similar to tissue extracellular matrices, which allows it to be used as a cell carrier. In this case, the mesh size is one of the foremost parameters for the efficient diffusion of substances, oxygen, and other things. Thanks to the proposed synthesis of a hydrogel based on two-phase aqueous emulsions of Alg and caseinate (Cas), crosslinked with calcium ions, the effect of the mesh size in the hydrogel on diffusion was evaluated. The average mesh size was varied from 97 ± 40 to 15 ± 8 μm by changing the volume ratio of the Alg and Cas phases. The study showed that hydrogels with the most prominent pores, measuring about 97 ± 40 µm, exhibit the lowest storage modulus values compared to other samples. It may mean that such hydrogels promote more efficient remodeling of cells encapsulated in them.

One work where porosity plays a critical role in the diffusion process is a study on producing stem cell spheroids for wound healing by a reversible porous hydrogel [165]. In this work, a group of scientists proposed to implement the formation of spheroids inside poly(*N*-isopropyl acrylamide) (PNIPAM) in situ. The three-dimensional network was formed by copolymerization of NIPAM with *N*,*N*′-Bis(acryloyl)cystamine (BAC) and allyl polyethylene glycol (APEG) to build a porous structure and impart hydrophilicity. The proposed structure solved the issue of low response rate due to the limited collective diffusion of the hydrogel after it breaks down above the lower critical solution temperature, as reported in many studies. In this study, porous hydrogel networks provided a fast response and improved diffusion by increasing the contact surface area between polymer and solvent. Due to its porous structure, the proposed material allows the formation of spheroids of fat cells inside the porous structure. It has the property of a reversible gel–sol–gel transition to implement the collection and fixation of spheroids to increase the rate of wound healing.

When fabricating porous scaffolds, it is principal to consider tortuosity, the migration path of a molecule or cell to penetrate the gel [166]. This parameter affects the permeability of 3D hydrogel scaffolds and is achieved by mimicking the complexity of natural architecture, which improves cell attachment, proliferation, and cell migration during tissue repair (Figure 3c). Regarding cells penetrating scaffold structures, permeability is a more appropriate term than tortuosity. Permeability can measure the mass transfer of the scaffold and evaluate its topology, thereby determining the effectiveness of hydrogel scaffolds [167].

Despite significant progress in tissue engineering, one of the unsolved problems is the limitation of mass transfer. Therefore, much attention is paid to the issue of revascularization in engineered tissues to avoid necrotic zone formation. By combining microfluidic networks with hydrogels, it is possible to enhance mass transfer within hydrogels and regulate the chemical microenvironment of cells in a spatiotemporal manner, reproducing the role of natural microvessels. This approach overcomes the limitations of diffusion, which can be slow and inefficient in large hydrogels. Microfluidic channels can also regulate the chemical microenvironment of cells by controlling the flow of nutrients, growth factors, and other signaling molecules. Reproducing the role of natural microvessels, microfluidic networks within hydrogels can create a more physiologically suitable environment for cells in vitro, which may improve their function and behavior.

Thus, hydrogels have the potential to revolutionize regenerative medicine [168,169,170,171,172]. They are biocompatible, biodegradable, and have several other advantages, making them a versatile tool for clinical use. Despite already being used in medicine, their design and synthesis are a current trend in this field. In particular, diffusion control is an essential aspect of the design of hydrogels. It allows the provision of a sufficient amount of the necessary substances to replace damaged tissues and accelerate the regeneration process successfully. The material is expected to become more critical in this area as the potentialities based on new knowledge increase.

## 6. Practical Application of Limited Diffusion Kinetics in Agriculture

**Figure 4:**
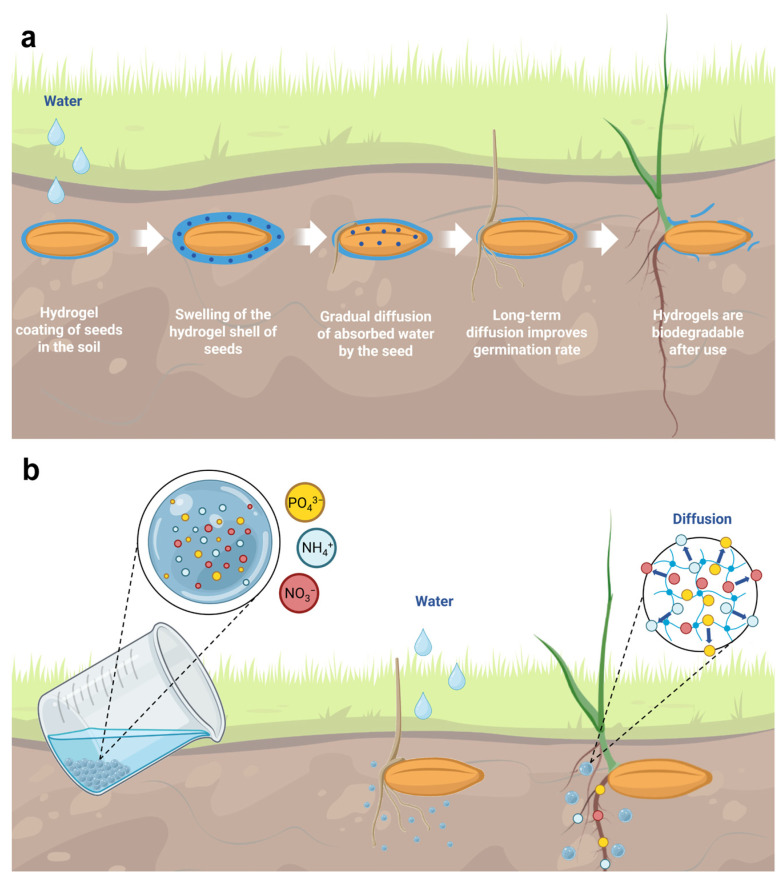
Approaches to hydrogels in agriculture: (**a**) Fertilizer encapsulation in hydrogels followed by soil application and diffusion mechanism. (**b**) The mechanism of hydrogel coating of seeds for constant moisture availability at the germination stage.

Agriculture is one more vast area for biocompatible hydrogels as a diffusive element. Moreover, this area opens up scope for development and requires well-reproducible low-cost solutions for industrial applications today. As for regenerative medicine, functional support in agriculture is useful and can be replaced entirely or absorbed after fulfilling its role. Controlled release in agriculture is often achieved by a simple uptake/desorption diffusion process from an area of high concentration to a place of low concentration. Substances useful for agricultural plants are encapsulated in hydrogels based on natural polymers due to their safety, biocompatibility, environmental friendliness, and ability to retain water and slowly release nutrients [173] (Table 2). Most often, pesticides [174], herbicides [175], fungicides [176], or fertilizers are encapsulated in hydrogels to enhance the protective or nutritional properties of the captured active ingredients [173,177] (Figure 4a). A separate important application of hydrogel in agriculture is the regulation of soil moisture by capturing and controlling the sustained release of water into the soil during dry periods [178].

Controlled release fertilizer (CRF) allows for efficient use of nutrients by reducing nutrient loss, thus minimizing environmental pollution. In addition, it is worth noting that using CRF increases the yield by 50% due to the slow release of conjugated agrochemicals from bifunctional lignin-based hydrogels due to the gradual cleavage of the ester bond [189]. At the same time, due to controlled release, it is possible to maintain the optimal concentration of agrochemicals for an extended period and, to a large extent, avoid losses during leaching [190]. The controlled release of urea in castor oil polyurethane-coated urea granules resulted in a 70% reduction in N_2_O emissions from the soil [191].

In a recent study, a superabsorbent resin synthesized by reverse suspension polymerization was modified with sodium humate as a fertilizer to promote plant growth [192]. The composite looked like poly(acrylic acid)/2-acrylamide-2-methyl propyl sulfonic acid/chitosan/sodium humate and is a homogeneous microsphere from 100 to 150 µm. The introduction of potassium persulfate as a free radical polymerization initiator in an amount of 1.2% and *N*,*N*-methylene bisacrylamide as a crosslinking agent in the range of 0.1–0.3% allowed achievement of the maximum water-absorbing capacity of the supersorbent (1096 g/g). Meanwhile, the water-holding capacity was good even under heating conditions and was 77.2%, 59.4%, and 52.9% at 30 °C, 50 °C, and 70 °C, respectively, for 5 h. The sodium humate in this system is chemically bonded to acrylic acid and chitosan, which are released from the superabsorbent resin due to a complex swelling–dissolution–diffusion process. In a similar study, a superabsorbent polymer was obtained by a simple chemical procedure of hybridization of chitosan and cellulose into one stable hybrid through chemical bonding using a thiourea–formaldehyde resin [193]. This hybrid can serve as an integral basis for the postgraft copolymerization of acrylic acid. The result was a new superabsorbent with improved reactivity, higher water absorption capacity (390 g/g), and increased mechanical strength compared to cellulose or chitosan alone.

Another study was aimed at synthesizing a superabsorbent hydrogel for the controlled release of water and urea by crosslinking sodium alginate (Alg) and *N*-(2-hydroxy-3-trimethylammonium) propyl chitosan chloride (HTACC) using calcium chloride [194]. The maximum loading of 5M urea was observed at a ratio of 70% Alg:30% HTACC and amounted to 200% due to surface multilayer adsorption, which resulted from adsorbate–adsorbate solid interaction. Urea was absorbed through single-layer and multilayer processes; on the first day, the release reached 20%. After three days, the cumulative release was 45%, and after 30 days, a release of 77% was achieved. The obtained superabsorbent hydrogel showed a slower release of urea than in other similar works [195,196], as well as biodegradation and good antimicrobial properties against several bacteria that can cause plant and human diseases. Thus, the temporal nature of urea release is crucial for many of the subsequent conversions of nitrogen in the soil.

The use of boron in agriculture is necessary for the average growth, development, productivity, and quality of crops due to its decisive role in metabolic processes [197]. The mechanism and kinetics of boron release from guar gum hydrogel were studied in [190]. The swelling of the resulting hydrogel, called boron-saturated superabsorbent hydrogel (BLSAH), increased from lower to higher pH in solutions, and the swelling index for pH 4, 7, and 9 was 20.7, 189.3, and 356 g/g, respectively. The ability of the hydrogel to absorb in an alkaline environment can be explained by the strengthening of hydrogen bonds and the expansion of the crosslinked network due to anionic repulsions, which promote the free penetration of the surrounding phase into the hydrogel [198,199]. The release of boron from the hydrogel occurs in three phases, with the first phase releasing about 60% of boron in 5 days due to matrix relaxation, followed by a moderate release phase from 2 to 6 h where boric acid dissolves in water and diffuses out, and a slow release phase from 6 to 120 h, when boron transfers from inner to outer layers via diffusion. Thus, BLSAH was found to have a maximum absorption capacity of 356 g/g and released 38% of the boron during a 30-day incubation period in soil, compared to 51% released by commercial boric fertilizer. Crosslinking was necessary to create a three-dimensional network structure in hydrogels, which allows for control over their mechanical properties, stability, and ability to retain water [200].

Crosslinking polymers is a common technique used to improve polymers’ mechanical and physical properties. Crosslinking creates a three-dimensional network of polymer chains linked together by covalent bonds, which can increase the material’s strength, stiffness, and thermal stability [201,202,203]. In the context of starch–chitosan hydrogels, the crosslinked polymer chains formed a three-dimensional network that restricted the movement of herbicide molecules, slowing down their release into the surrounding environment [204]. In turn, the degree of crosslinking and the choice of crosslinking agent play a role in controlling the release rate. For example, hydrogels with glutaraldehyde as a crosslinking agent showed a slower herbicide rate release than those with glyoxal. Overall, polymer crosslinking has been a leading factor in achieving controlled release properties of fertilizers in a few studies [205,206,207].

The promising use of controlled release hydrogels has shown itself as seed coats in the early stages of plant germination [208,209]. By enveloping the seeds with hydrogel, water and nutrients can be available during dry periods, which improves seed germination (Figure 4b). The hydrogel coating absorbs water by utilizing osmotic pressure generated by free ions and intermolecular repulsion between fixed ionic groups when free ions diffuse outward. However, the presence of salt or ions in the external solution reduces the potential difference, restricting the outward diffusion of free ions. Consequently, hydrogel absorbs a lesser amount of solution. A hydrogel coating of succinate-modified potato starch was found to positively affect the early growth of corn seeds under conditions of moderate moisture deficiency [210]. Due to the long water retention of the hydrogel and the slow release near the seed, the seeds can continue to absorb water even when the soil dries out.

The application of hydrogel alginate containing fertilizer nutrients, plant growth biostimulants, and amino acids derived from high-protein material to the surface of the seeds ensures slow release of nutrients, high bioavailability of fertilizer nutrients, and low leaching of nutrients into water [211]. The hydrogel coat encapsulates the nutrients and acts as a barrier, delivering them through a controlled release mechanism. Micronutrients serve as a crosslinking agent for the coating and diffuse from the hydrogel matrix in a controlled manner [212]. Pot tests on cucumbers confirmed the new method’s effectiveness, yielding a 50% higher fresh sprout weight and four times greater root length than uncoated seeds.

Thus, hydrogels have the potential to revolutionize agriculture by improving water and fertilizer release, increasing soil porosity, and addressing water scarcity. However, further research is needed to optimize synthesis and application methods, ensure biodegradability and affordability, and seek regulatory approval for commercialization.

## 7. Practical Application of Limited Diffusion Kinetics in Soft Robotics and Microrobotics

Despite a significant preponderance towards applications in medicine and other near-biological fields, hydrogels are also used in unexpected areas closer to mechanics or computing. Hydrogels are currently utilized as pliable materials for soft robots and as soft sensors and actuators due to their ability to respond to various stimuli such as temperature, pH, and light.

Drug delivery self-propelled vehicles, often named nano- or microswimmers, are small devices that can navigate the body to deliver drugs to specific sites. Hydrogels have been explored as a material for drug delivery microswimmers due to their ability to encapsulate and release drugs in a controlled manner [213,214]. These microswimmers are propelled by various mechanisms, such as magnetic fields, acoustic waves, or chemical gradients [215]. Hydrogel-based microswimmers have shown promise for applications in targeted drug delivery, as they can navigate through complex environments and reach specific cells or tissues. While nano- and microgels rely on passive diffusion or stimuli-responsive release, as discussed previously, nano- and microswimmers migrate by diffusion-driven mechanisms. Autonomous movement of hydrogels typically refers to nano- and microgels, which have specific catalytic or active units allowing their self-propelled transportation to the targeted site via external stimuli. Previously discussed diffusion-mediated mechanisms of release are all applied to the smart nano- and microswimmers [216]: they are dissolved [217] or swollen [218] to release the drug and biodegrade when the task is accomplished. The common environmental triggers allow guidance of self-propelled vehicles via chemical or physical stimuli. Self-diffusiophoresis, one of the self-propelling mechanisms of the nano- and micromotors decorated with a catalytic species, defines the drug carrier’s movement forward in the physiological environment [217]. Self-generated concentration gradients by fuel degradation at the catalytic side of motors provide an inhomogeneous distribution of molecules around particles and induce a directed migration of vehicles [219]. Therefore, the diffusion is limited by the presence of the fuel, which propels the particle. Currently, developed polymeric nano- and micromotors for in-body drug delivery are capable of utilizing physiological fluids as fuel for converting chemical energy into motion: hydrochloric acid in gastric juice [220], glucose in blood [221], hydrogen peroxide at inflammation and tumor sites [222], or urea in blood [223]. Another diffusion-guided mechanism, chemotaxis, was also observed for nano- and micromotors. This process occurs in nature among cells or bacteria, which sense the environment and migrate in response to chemical gradients, either for nutrition purposes or to escape harmful conditions [224]. The reason behind the chemotaxis behavior is linked to the increased active diffusion coefficient at elevated levels of hydrogen peroxide or other fuel. Chemotactic behavior defines the precision of localized drug delivery processes as the particles move towards the source of the fuel, finding the disease site which produces it, for example, a hydrogen peroxide-releasing tumor or inflammation [225].

Diffusion reactions are often accompanied by swelling/deswelling, pH changes, hydrophobic/hydrophilic transition, etc. Such changes can be used for soft programmable electronics. In the present section, we focus on hydrogels made with expansion/contraction, swelling/deswelling, and the presence of inorganic conductive parts’ properties of soft materials for an electronic application. However, the power supply for these materials is still one of the most significant challenges, restricting the application of soft electronics. Hydrogels could be used for creating flexible supercapacitors, whose properties are maintained with the mechanical transformation of material. In soft robotics development, hydrogels are attractive due to stimulating water diffusion for swelling and deswelling, providing volumetric changes without external stimulus [226]. Diffusion and osmotic pressure limit the natural response of the body by hygroscopic expansion/contraction. Scientists successfully embedded plants’ physiological features in hydrogel applications [227,228,229]. A solution diffusion model could describe limited diffusion in hydrogels, whereas polymer together with solute is a molecular mixture and solute transport is provided by diffusion.

The other most prominent feature of studies on soft electronics systems is that they can change electronic properties correlated with mechanical stretchings [230,231,232]. Hydrogels for soft actuators have been widely studied by Velev’s group [233,234,235,236,237]. Researchers constructed “soft robot” prototypes uniting electronics, polyelectrolyte hydrogels, and biomimetic structures. They focused on the system to transfer chemical energy to mechanical properties without external stimuli. Soft biocompatible materials will be applied to tissue scaffolds, biomedical materials, 3D printing, and flexible electronics. Due to demanding mechanical self-supporting and load-bearing features, soft programmable electronic devices needed a high Young’s modulus and compression of biomedical tissue [238].

The two most notable groups of soft programmable electronics are separated by the main electrical element inside. The first are conducting polymers [239,240,241], whose properties do not demand other support and are enough to obtain the electrical signal from the material. The widely used polymers are PEDOT-based assemblies [242,243,244,245,246], polypyrrole [247,248,249,250], and acrylamide copolymers [239,251,252,253].

Charged macromolecules—polyelectrolytes—have become widely applied in soft electronics [254,255,256]. Charge redistribution provides a tuning possibility for polyionic complexation between polyelectrolytes to convert the gel network to a rigid structure. The method’s restriction reveals the viscosity of the polymer suspension leading to poor mechanical properties and swelling behavior. However, using polyelectrolytes in soft programmable electronics is still favorable due to the anisotropic effects of macromolecules and biomimetic properties. Also, DNA has become widespread for programmable electronics due to its biocompatibility and ease of further in vivo investigation [241,257,258]. For DNA, the main problem is the low interfacial connection which maintains the level of interactions.

**Figure 5:**
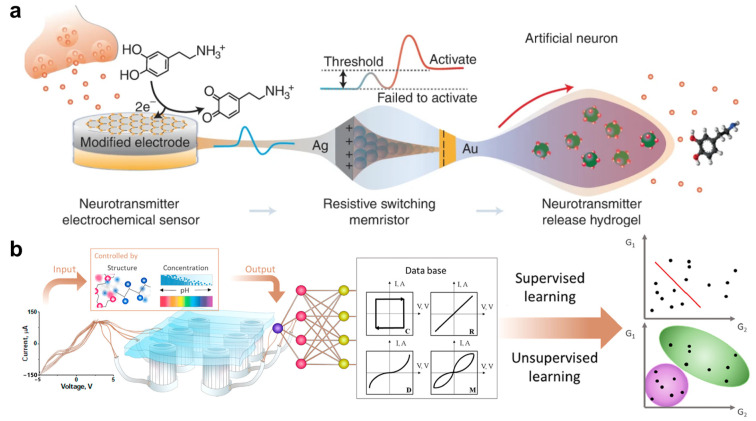
(**a**) Scheme of neurotransmitter-mediated artificial neuron modulating a typical memristor device; CC: Compliance current; HRS: High-resistance state; LRS: Low-resistance state [274]. (**b**) Prototype of a chemical perceptron based on polyelectrolyte hydrogel with four types of electronic behavior and machine learning analysis [277].

On the other hand, the second common approach is integrating materials into the hydrogel matrix conductive parts, such as 2D materials [259,260,261], semiconductors [262,263,264], liquid crystal [265,266,267,268], and MXene [250,269,270,271,272,273]. Several different types of electrical behavior can be achieved by hydrogel materials, two of which are represented in Figure 5. The diode features decrease with the increasing mechanical frequencies by changing the diffusion characteristics of the presence of ions. The diode behavior of the soft system supposes an application for a prototype of sensor arrays. Furthermore, hydrogels can be a part of artificial neurons to implement electrochemical properties in the sensor signal system (Figure 5a). Ting Wang, with coauthors, designed an artificial synapse with a sensing memory and memristor constituants [274]. Ag nanoparticles were chosen to achieve electrical properties and transfer sensor signals by memristor behavior with synaptic plasticity. Depending on the electrical stimulus, a memristor can adapt its internal resistance for the next step. The hydrogel is compressed after memristor resistance reaches a target value, releasing the main compound. Soft programmable hydrogels with diffusion mechanisms can switch electrochemical behavior by external stimuli [275,276]. Ivanov et al. [277] presented a model to change electrical behavior by voltage (Figure 5b). This effect dramatically depends on including gallium ions in polyelectrolyte-based hydrogel under the applied current. Different compositions of hydrogels provide a change in intensity and lead to current redistribution in the circuit. Therefore, the authors suggested a self-switchable soft electronic chemical system that can achieve four types on behavior: capacitor, diode, memristor, and resistor by controlling the interface of oxide thin films.

Zhang Yung et al., established a hydrogel ionic diode based on layered anionic and cationic compounds with immersed carbon nanotubes (CNTs) and silver nanowires (AgNWs) to provide analogues of p-semiconductor junctions [278]. Inorganic parts gave a distinguished reflection ratio, leading to promising mechanical energy-harvesting characteristics. Transistor sensors are one of the most promising applications of hydrogels as soft programmable electronics. Jin Hu et al. [279] suggested an adaptive platform for developing hydrogel-gated devices for multifaceted applications. The elaborated organic photoelectrochemical biosensing transistors presented a short detection time and sensitive detection performance. The hydrogel-gated system operated by Ca^2+^ triggered gelation for further coupling with a PEDOT:PSS channel. The light-generated photovoltage on the interface operates the device; therefore, the hydrogel gating effect was achieved. In general, the application of soft programmable hydrogel is mainly focused on wearable electronics with a balance of charge capacitance and lower materials’ cost. L.T. Duy and H. Seo [280] combined the stretchable properties of hydrogel and capacitance behavior to suggest a wearable soft programmable electronic system prototype. Reduced graphene oxide was used as a base for hybrid hydrogel mixing with polymer compounds. Electrochemical measurements proved the supercapacitance properties of developing materials. Surprisingly, the increase in supercapacitance was low upon the stretching, which was attributed to domain redistribution in hydrogel along the stretching direction.

Soft programmable hydrogels find applications for creating logic gates. Stimuli-responsive hydrogel provides several valuable properties, such as biocompatibility, the possibility of designing the shape of the devices, and the programming of output by an applied Boolean logic-based algorithm [281]. The group of Prof. DeForest created a biomimetic soft system, which controls properties by molecular architecture and ordering of stimuli parts [282,283]. The logic gates are based on the two degradable linkers, which assemble depending on the environmental inputs. Release of the model therapeutics occurs according to Boolean logic: an OR response provides usage of target delivery; by comparison, the AND-gated system leads to increased target specificity. The researchers suggested investigating systems for bioapplications, especially cancer treatment.

Another example of hydrogel-based logic gates is the implementation of enzyme reactions in the soft matter. Xinlong Fan and Andreas Walthe [284] prepared a multienzyme hydrogel sensor with a biocompatible mimic of living equilibrium conditions. Using an enzymatic cascade system, separating a YES gate and NAND gate depending on pH signals from a different optical form of glucose oxidase chain was possible. The NAND gate was suggested as an enzyme cascade until the YES gate was implemented by adding Tirs buffer to achieve a transient signal. Different pH-responsive mechanisms could be interesting observations for fabricating new self-oscillation reaction networks.

Therefore, there is no problem developing functional materials based on the self-assembled structure to observe the accurate response from the coupling of stimuli (external or internal) with an output signal which has predicted the autonomous life cycles of soft matter.

## 8. Future Prospectives

Hydrogels are a broad class of materials known since ancient times and have not lost their relevance in modern times. The main fundamental works on diffusion in hydrogels were written quite a long time ago. However, this does not prevent the search for applications for hydrogels and their use in related fields.

Currently, hydrogels are primarily associated with medicine, food, and agriculture. Major scientific advances are also associated with these fields. Given the size of the research groups, this trend will remain in medical research, which is in good agreement with the trends in improving the standard of living and individualized medicine. Thus, we should expect discoveries in targeted drug delivery by diffusion release from hydrogels. Moreover, two growing areas are changes in the form factor and architecture of hydrogel devices and matrix functionalization.

The use of hydrogel scaffolds in regenerative processes, even more important from the point of view of medicine, is also primarily based on the processes of diffusion and replacement. Here, we should expect breakthroughs in the issues of long-term biocompatibility and biomimetic support of cell proliferation using the spatial organization of hydrogels assembled from biomaterials.

In agriculture, we should expect an increase in the number of approaches to the individualization of plant management to reduce the number of fertilizers and save resources, which is also consistent with modern environmental trends. It will require the development of cheap biodegradable hydrogels as well as the development of cheap routes for their targeted delivery.

In addition, ion-conducting materials in the energy sector contribute to new areas that will also be widely developed. Here, their ability to expand the temperature limits of the applicability of liquid elements and their protective and mechanical performance characteristics will be used. The same feature is also utilized in a more complex area of soft electronics; however, the primary results are too scarce for predictions.

Moreover, it is worth noting the area of directed diffusion control in chemistry, which allows growing materials with desired spatial and structural characteristics, as well as the growing trend towards the creation of ultraselective sorbents based on hydrogels, including functionalized biomolecules such as proteins or synthetic DNA.

## 9. Conclusions

Hydrogels have immense potential in various fields, such as regenerative medicine, drug delivery, and agriculture, and their unique properties make them ideal for use in bioelectronics. Hydrogels have numerous advantages in drug delivery, such as biocompatibility and good swelling behavior. Limited diffusion in hydrogels helps to ensure controlled release, maintain desired properties, and preserve the structural integrity of the hydrogel. However, limitations exist depending on the gel-forming polymers’ chemical moieties and the administration route. Overcoming these limitations requires a thorough understanding of the physiochemical properties of the polymers and the influencing factors that control their behavior. Once a general understanding is reached, the efforts split into fine-tuning the properties and practical applications for the materials in the lab and on an industrial scale. In agriculture, hydrogels have effectively protected soil and improved plant performance under harsh environmental conditions. Due to limited diffusion in hydrogels, it is possible to regulate the release of fertilizers into the soil for a long time. However, nonbiodegradable superabsorbent polymer hydrogels may be toxic and not produced from renewable materials. Natural hydrogel-based agents have demonstrated positive seed and plant protection results without compromising soil fertility, water consumption, or nutrient loss. The unique properties of hydrogels are a precise regulation of interfacial mass and transport, creating new opportunities for biosensor and biocatalyst development. Recent advancements in stretchable, biodegradable, self-healing, and bioadhesive hydrogels offer even more significant potential for designing bioelectronic interfaces with intimate contact and minimal invasiveness. With continued research and development, hydrogel-mediated biointegrated electronics will play a vital role in disease diagnosis and personalized medicine. Overall, hydrogels offer a promising avenue for developing sustainable solutions in various fields, but further research and development are necessary to overcome existing challenges.

## Figures and Tables

**Table 1:** Design of criteria for controlled diffusion in hydrogels.

Type of Hydrogel	Strategies for Changing the Diffusion Rate	Application	References
Composite nanofibrous scaffolds of polyamide-6, polyvinylpyrrolidone, and tea tree oil	Molecular weight of the polymer	Obtaining nanofibers for the manufacture of antimicrobial wound dressings	[153]
Chitin whisker/chitosan (CHW/CS) hydrogels	Crosslinking density	Dual-crosslinked liquid crystal hydrogels are good candidates for bone repair applications	[154]
Hyaluronic acid nanocomposite hydrogel (HA-BP hydrogel) by coordination bonds with bisphosphonates (BPs)	Doping of metal	Highly dynamic nanocomposite hydrogels that self-assemble by coordinating metal ions and ligands enable new dynamic materials for regenerative medicine	[155]
Nanocomposites of silver nanoparticles/gelatin	Thermoresponsive	Silver diffusion-controlled hydrogel is a tool as an effective dosage form for topical wound healing	[156]
The functional hydrogel encoding the binding domain of laminin (Fmoc-DDIKVAV)	Addition of myoglobin	The hydrogel can control cell fate in progenitor cell grafts, enabling the successful integration of stem cell grafts to treat nerve damage and illnesses affecting the central and peripheral nervous system	[157]

**Table 2:** The application of natural polysaccharide-based hydrogels for agriculture.

Types of Hydrogel	Crosslinking Agent	Included Fertilizer	References
Alginate–cellulose nanofibers–PVA	Calcium sulfate	Potassium chloride, ammonium dihydrogen phosphate	[179]
CS–alginate	Copper sulfate	Trichoderma viride, copper cations	[180]
CMC–CS	Manganese sulfate	Prothioconazole	[174]
CS	Sodium tripolyphosphate	Hexaconazole, dazomet	[181,182,183]
CMC-g-poly(AM-co-AMPS)	*N*,*N*’-methylenebisacrylamide	2-Chloroethylphosphonic acid	[184]
MS-g-PA	*N*,*N*′-methylenebisacrylamide	ZnO/tetraethyl orthosilicate	[185]
Starch–cellulose	*N*,*N*′-methylenebisacrylamide	Urea	[186]
Starch–PVA	Acrylic acid, citric acid, and maleic acid	Urea	[187]
Carboxymethyl starch/polydopamine	Monochloroacetic acid	NH_4_^+^, Zn, P, and Fe	[188]

CMC: Carboxymethyl cellulose; CS: Chitosan; AM: Acrylamide; AMPS: 2-Acrylamido-2-methylpropanesulfonic acid; MS: Modified starch; AA: Acrylic acid; PA: Polyacrylate; PVA: Polyvinyl alcohol.

## Data Availability

Not applicable.
